# Corrosion Behavior of Cold-Formed AA5754 Alloy Sheets

**DOI:** 10.3390/ma14020394

**Published:** 2021-01-14

**Authors:** Anna Dobkowska, Agata Sotniczuk, Piotr Bazarnik, Jarosław Mizera, Halina Garbacz

**Affiliations:** Faculty of Materials Science and Engineering, Warsaw University of Technology, 02-507 Warsaw, Poland; agata.sotniczuk.dokt@pw.edu.pl (A.S.); piotr.bazarnik@pw.edu.pl (P.B.); jaroslaw.mizera@pw.edu.pl (J.M.); halina.garbacz@pw.edu.pl (H.G.)

**Keywords:** AA5754, corrosion, microstructure

## Abstract

In this work, the influence of bending an AA5457 alloy sheet and the resulting microstructural changes on its corrosion behavior was investigated. Scanning electron microscopy (SEM) and transmission electron microscopy (TEM) were used to perform detailed microstructural analyses of the alloy in its original form and after bending. After immersion in naturally-aged NaCl under open-circuit conditions (0.5 M, adjusted to 3 by HCl), post-corrosion observations were made, and electrochemical polarization measurements were performed to investigate the corrosion mechanisms occurring on both surfaces. The results showed that the corrosion of AA5457 is a complex process that mainly involves trenching around coarse Si-rich particles, crystallographically-grown large pits, and the formation of multiple tiny pits around Si-rich nanoparticles. The experimental data showed that bending AA5457 changed the shape and distribution of Si-rich coarse particles, cumulated a higher dislocation density in the material, especially around Si-rich nanoparticles, and all of these factors caused that corrosion behavior of the AA5754 in the bending area was lowered.

## 1. Introduction

Due to their high mechanical and anti-corrosion properties, low density, and good formability, the 5xxx series of Al alloys is widely used in many branches of industry, particularly in the automotive industry [1,2,3,4,5,6]. Their chemical composition mainly includes Al and Mg (up to 7 wt.% Mg), along with secondary additions of Mn and trace amounts of Cr [7]. Their corrosion resistance arises from a complex combination of factors, but it strongly depends on the microstructural features of the alloy [8,9,10]. Several studies have reported the role of intermetallic particles in the corrosion resistance of Al alloys, and these particles can be organized by their size and electrochemical behavior with respect to the Al matrix [11,12,13,14,15,16]. If the particles are relatively large (a few µm), they are classified as coarse particles, but nanoprecipitates also often form in Al-Mg alloys. Due to their various electrochemical properties (anodic/cathodic), they can either suppress matrix dissolution or have only a small influence on the corrosion of Al alloys [17,18]. Particles rich in Zn or Si (such as MgZn_2_ or Mg_2_Si) are prone to dissolution [19]. Generally, Fe-rich particles, i.e., Al_n_ (Fe, Mn), are nobler with respect to the Al matrix and decrease the localized corrosion resistance of Al alloys depending on the content of other impurities [20,21,22]. The local corrosion attack around intermetallic particles has been explained in terms of various particle behaviors in corrosive media: particle fall-out, selective particle dissolution or particle dealloying [19,23]. Recent works on the microstructure of alloys have revealed different types of coarse particles in Al-Mg alloys, depending on the additional alloying elements.

The second type of local attack also occurs at small precipitates formed in 5xxx series Al alloys. When the Mg content exceeds 3.0 wt.% and alloys are exposed to temperatures of 50–225 °C, Mg atoms preferentially diffuse from the supersaturated solid solution (α) to grain boundaries (GBs). Then, the variety of Mg-rich phases changes from metastable (β′ and β′′) to equilibrium β (Al_3_Mg_2_) precipitates at GBs (sensitization) [24,25,26]. Al_3_Mg_2_, which behaves as an anode with respect to the Al matrix, plays a predominant role in the corrosion resistance of Al-Mg alloys [19,27,28,29]; however, due to its small size (~100 nm or smaller), it is difficult to characterize this phase in situ [30,31]. The quantitative detection of the dissolution of β precipitates regarding the corrosion behavior of 5xxx alloys has been thoroughly studied by Guan et al. [25]. Vignesh et al. [32] analyzed the susceptibility to intergranular corrosion (IGC) of AA5083 and showed that the alloy’s susceptibility to IGC may be reduced by grain structure refinement, dispersion, and the partial dissolution of secondary Mg_2_Al_3_ in the matrix. This has been confirmed in another study [33], which showed that the pitting, IGC and exfoliation all depended on the precipitate distribution. The study suggested that grain refinement can effectively enhance the IGC resistance of the commercial AA5083-H111. Moreover, the selective dissolution of Al_3_Mg_2_ phase in AA5083 also determines its resistance to stress corrosion cracking [34]. In 5xxx Al alloys, pitting is explained in terms of the formation of a defective oxide film [35] and is strongly dependent on the NaCl concentration and pH of the solution [36,37,38].

Since the 5xxx alloys do not respond to age hardening, previous reports regarding the electrochemical behavior of 5xxx alloys have also focused on post-processing heat-treatment and/or plastic deformation [39,40,41]. As stated by Neetu et al. [42], the subsequent annealing of the AA5080 produced by multi-axial forging at room temperature drastically improved its corrosion resistance. The homogenization heat treatment of AA5083-O plates after casting had the optimal effect on the overall corrosion resistance of the material [40]. Various plastically-deformed (i.e., friction stir processing, accumulative roll-bonding) Al-Mg alloys have shown better corrosion resistance compared with their parent material [43,44]. In contrast, extruded AA5083 showed similar overall corrosion resistance compared with conventional Al-Mg alloys [45].

Clearly, the corrosion behavior of Al-Mg alloys is complex and requires more studies to distinguish the dependence between the formation of various kinds of localized corrosion and their mutual interactions. Although Al-Mg alloys display fairly good corrosion resistance [46,47], subjecting them to additional cold-forming to produce semi-products or final elements may change the material’s properties and compromise their electrochemical behavior. Therefore, the optimization of the corrosion performance of commercially applicable Al-Mg alloys is of critical importance in terms of using these alloys for structurally lightweight devices and when plastic deformation is necessary to obtain the desired shape of a design element (i.e., bending). For the needs of this project, we produced part of a construct using AA5457, which has an optimal combination of mechanical properties and is lightweight, commercially available, and prone to plastic deformation. In this work, Al-Mg type AA5754 alloy was used to produce the outer components of an experimental prototype of a capsule that allows for teleperformance assessment tests and telerehabilitation of sensory diseases (i.e., hearing, sight, smell, balance). The main goal of this study was to analyze how cold-working can change corrosion resistance of AA5754 (O/H111 according to [48]).

## 2. Materials

**Figure 1:**
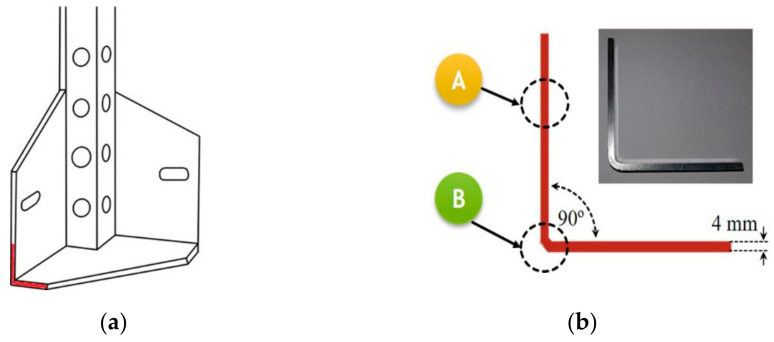
The outer design of the transferable capsule used for teleperformance assessment tests and telerehabilitation: (**a**), schematic of the outer elements, and (**b**), locations of corrosion tests.

A schematic of the construction design is given in Figure 1a. To prepare a final element in the form of an adjustable angle plate, a 4 mm-thick sheet made of AA5754 (chemical composition given in Table 1) was cut into strips and subsequently bent to 90° (Figure 1). The main focus was to determine the influence of microstructural changes induced by bending on the corrosion behavior of the original AA5754 sheet. To accomplish this, the corrosion behavior in two specified areas of the final element was measured: in the undeformed area located at the arm of the bent Sheet (**A**) and at the bent area (**B**), as shown in Figure 1b.

## 3. Methodology

### 3.1. Microstructure Characterization

Microstructure characterization was performed in two areas (undeformed and bent areas) using scanning electron microscopy (SEM, Hitachi SU8000, Tokyo, Japan) in back-scattered electron mode (BSE) with an accelerating voltage of 5 kV. The microstructure was observed on the cross-section of the Sheet in areas A and B shown in Figure 1b. Specimens for SEM observations were prepared by grinding samples with up to 1200-grit SiC paper and ion polishing with a Hitachi IM-4000 ion milling system. The ion milling is a damage-free process that does not cause surface deformation, eliminates stresses and oxide layers; therefore, the surface quality is good enough to observe the structure under channeling contrast using SEM. Structural investigations were combined with an energy dispersive X-ray (EDX) analysis of precipitates and intermetallic particles. To more thoroughly describe the role of intermetallic particles, the density of coarse particles was calculated by dividing the number of counted particles (*N*) by the studied area (*A*) of the specimen. Moreover, their size (*A*—area) and standard deviation (*S*_D_) were calculated using computer software as per references [49,50].

Detailed microstructural observations of selected areas were performed using a transmission electron microscope (TEM, JEOL 1200, Tokyo, Japan) operating at an accelerating voltage of 120 kV, and the EDX spectra of the nanoparticles were obtained using a high-resolution scanning transmission electron microscope (STEM equipped with an EDX spectrometer, Hitachi S5500). The TEM samples were prepared using twin-jet electropolishing at 3 °C/20 V with an electrolyte composed of perchloric acid (HClO_4_), ethanol and 2-butyxoethanol (type A2 purchased from Struers, Ltd., Ballerup, Denmark) Based on the observations, the dislocation density (*ρ*) was calculated using the formula [51]:

(1) $$\rho =\frac{N}{L_{r}h}$$

where *N* is the number of dislocation-line intersections, *L_r_* is the total length of all lines, and *h* is TEM sample thickness (*h* = 150 nm).

### 3.2. Corrosion Tests and Characterization of Corroded Surfaces

Electrochemical tests were performed in an aerated 0.5 M NaCl acidified to pH 3 by the addition of hydrochloric acid (HCl) as per reference [52]. The electrolyte was made from analytical-grade reagents and distilled water. A FAS1 Gamry potentiostat (Gamry Instruments, Warminster, PA, USA) was equipped with three electrodes: Pt as the counter electrode, Ag/AgCl as the reference electrode (Ag/AgCl wire immersed in a saturated KCl solution), and the measured sample as the working electrode. The corrosion potential (*E*_corr_) was recorded during 3 or 6 h of immersion under open-circuit conditions. After immersion, the corrosion rate of samples was determined using linear polarization resistance (LPR), and potentiodynamic tests were conducted. LPR data were recorded over a potential range of *E*_corr_ ± 10 mV at a scan rate of 5 mV/min. After LPR, the samples were conditioned for 3 min and potentiodynamic tests were performed at a scan rate of 5 mV/s, starting from −0.5 V/OCP to 2 V/REF at room temperature. To prevent noise, all measurements were conducted in a Faraday cage. The polarization curves were fitted using Gamry Elchem software (Gamry Instruments, Warminster, PA, USA) in Tafel mode. To ensure the reproducibility of the results, all measurements were repeated at least three times on the same samples. The surfaces of the samples after immersion under open-circuit potential were observed using a Hitachi SU8000 SEM.

## 4. Results

### 4.1. Microstructure Characterization

**Figure 2:**
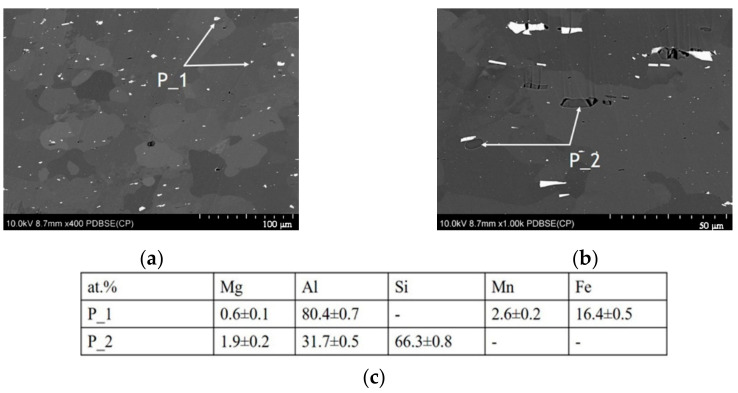
SEM images of the microstructure of the AA5457 sheet in the undeformed area (A): (**a**), an image showing the overall distribution of intermetallic particles, (**b**), image showing the morphology and surrounding area of intermetallic particles, and (**c**), chemical analysis of the intermetallic particles P_1 and P_2 marked in panels a and b.

**Figure 3:**
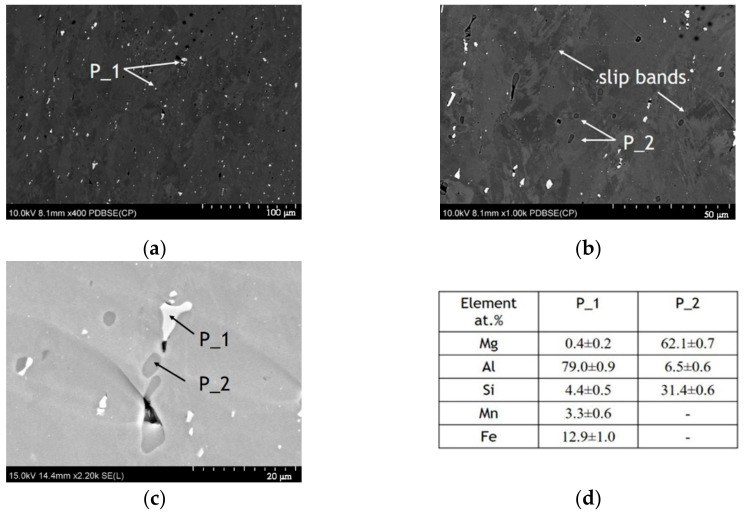
SEM images of the microstructure of the AA5457 sheet in the deformed area (B): (**a**) and (**b**), images showing the overall distribution of intermetallic particles, (**c**), image showing the morphology and area surrounding the intermetallic particles P_1 and P_2, and (**d**), the chemical analysis of the intermetallic particles P_1 and P_2 marked in panel c.

Figure 2 and Figure 3 show BSE images of the AA5754 alloy in an undeformed area and a deformed area, respectively. The BSE image shown in Figure 2a at a lower magnification shows a typical granular microstructure with many large, irregularly-shaped intermetallic particles randomly distributed in the alloy. The SEM-EDS analysis reveals that there are two kinds of particles: the white particles marked as P_1 are Al, Mg, Mn, and Fe-rich particles (Figure 2a,c), and the dark grey precipitates P_2 are composed of Mg, Al, and Si (Figure 2b,c). In the deformed region (Figure 3), the subsequent bending altered the microstructure of the material, and the distribution of the intermetallic particles also changed; more coarse white precipitates were observed in the bending area (Figure 3a). Additionally, during deformation, a significant strain was introduced into the material and resulting from this, higher dislocation density occurred [42]. Therefore, a granular structure is not clearly distinguishable in the BSE contrast image; however, many dislocations, especially multiple slip bands, are visible (Figure 3b). Similar to the undeformed area, the coarse intermetallic particles shown in Figure 3c marked as P_1 are mainly enriched in Mg, Al, Mn, and Fe (Si was detected in the bending area), while those marked as P_2 are composed of Al, Mg, and Si (Figure 3d).

The density of P_1 particles in the undeformed and deformed areas was comparable, *N*_A_ = 0.01 and *N*_A_ = 0.02 (given in 1 × µm^−2^), for the undeformed and deformed areas, respectively; however, after deformation, they showed smaller sizes (Table 2). The SEM images indicate that there was an abrupt change in the density of P_2 particles before and after deformation (compare Figure 2a and Figure 3a). The calculations demonstrate that the density of the P_2 particles changed to *N*_A_ = 0.001 and *N*_A_ = 0.01 (given in 1 × µm^−2^) in the undeformed and deformed areas, respectively (Table 2). As a result of bending, the observed particles were slightly smaller in size (Table 2).

**Figure 4:**
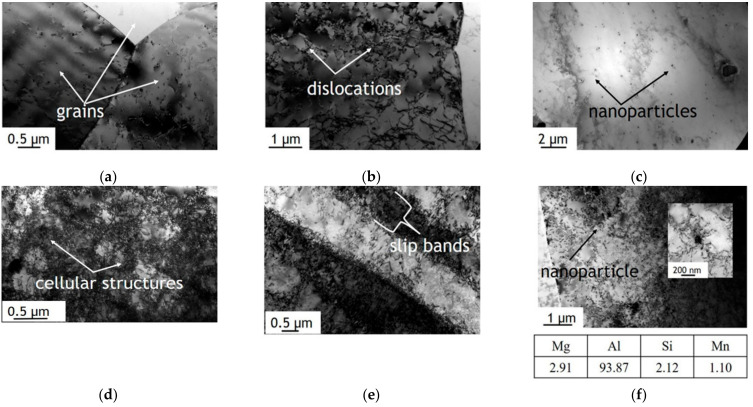
TEM images of the microstructure of the AA5457 sheet in the undeformed area (A): (**a**,**b**), images showing the overall grain boundaries and dislocation structure, and (**c**), image showing the overall distribution of nanosized intermetallic particles. TEM images of the microstructure of the AA5457 sheet in the deformed area (B): (**d**,**e**), images showing the increase in the dislocation density and the formation of cellular dislocation structures and slip bands, and (**f**), image showing an increase in the dislocation density around nanosized intermetallic particles. The representative chemical composition of a nanoparticle is presented below panel (**f**).

SEM structural analysis was complemented by TEM observations. Figure 4 presents TEM images of the undeformed (Figure 4a–c) and deformed regions (Figure 4d–f). The coarse grains (Figure 4a) contained only a few dislocations in the grain interior (Figure 4b). TEM investigations also revealed the presence of small uniformly-distributed nanoparticles, as illustrated in Figure 4c. The observed particles have sizes from 50–200 nm and are rich in Si and Mn (the chemical composition is presented below Figure 4f).

The results from the deformed region clearly show an increase in the number of dislocations compared with the undeformed section. The dislocation density increased by nearly an order of magnitude, from 4.5 × 10^12^ m^−2^ in the undeformed region (Figure 4b) to 4.4 × 10^13^ m^−2^ in the deformed region (Figure 4d,e). This is a typical phenomenon for deformed metallic materials. It should be noted that dislocations are distributed rather uniformly; however, in some regions, the formation of characteristic cellular structures (Figure 4d) and slip bands (Figure 4e) was observed. Moreover, an increase in the dislocation density was also observed around Si and Mn-rich nanoparticles (Figure 4f). Hard intermetallic particles pin dislocations during deformation, resulting in the formation of many dislocations around such particles.

### 4.2. Corrosion Testing

#### 4.2.1. Electrochemical Testing

**Figure 5:**
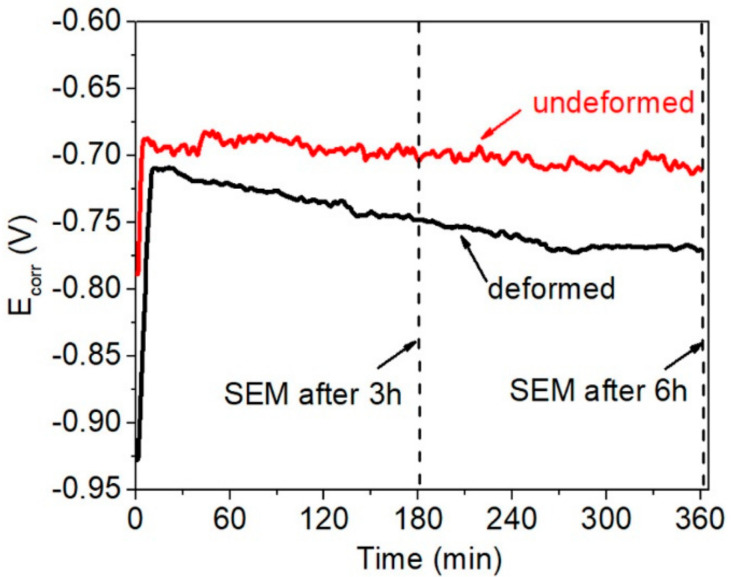
The *E*_corr_ measurements recorded during 6 h of immersion in 0.5 M NaCl (pH 3) in the undeformed and deformed areas of AA5754. SEM observations were made after 3 and 6 h of immersion, as marked by the dashed lines.

*E*_corr_ measurements recorded in naturally-aerated 0.5 M NaCl adjusted to pH 3 are shown in Figure 5. The evolution of *E*_corr_ in both samples exhibits a similar trend, with a rapid initial increase followed by a longer-term stabilization. The *E*_corr_ of the undeformed sample started at −0.78 V/REF, and increased to around −0.70 V/REF after the first 5 min of immersion. It maintained a near steady-state value of −0.70 V/REF for the remainder of the experiment. The initial increase of *E*_corr_ in the deformed area began from −0.94 V/REF and reached −0.72 V/REF after 10 min immersion. Then, a small but stable decline in *E*_corr_ to −0.78 V/REF was observed at the end of the experiment. The initial rapid increase in *E*_corr_ for both alloys may be related to the extensive corrosion and passivation processes [53], while the slight fluctuations observed in the *E*_corr_ curves were attributed to the pitting process caused by the constituents formed in the alloy [54,55,56].

**Figure 6:**
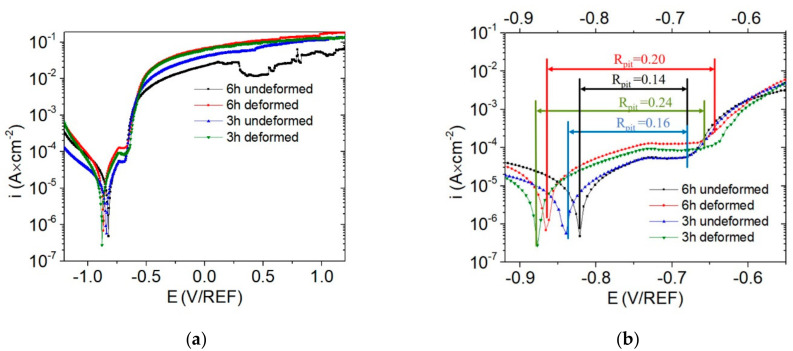
The electrochemical curves obtained after 3 and 6 h of immersion in 0.5 M NaCl (pH 3) for undeformed and deformed areas of the AA5754: (**a**), potentiodynamic polarization curves, and (**b**), pitting susceptibility of analyzed materials calculated using Formula (1).

The potentiodynamic polarization curves recorded for the undeformed and deformed samples of AA554 after 3 and 6 h of immersion under open-circuit conditions are given in Figure 6. All curves exhibit similar trends with passive regions, which suggests an anodic control of the corrosion processes. All curves exhibit a wide passive region when the current density plateaued, extended by 0.04 V in the undeformed area (both 3 and 6 h immersion), to around 0.05 V for the deformed area after 3 h of immersion, and 0.07 V for the deformed area after 6 h of immersion [57] Simultaneously, there is a shift in the pitting current, *i*_p_, which is the lowest for the undeformed area (after both periods of immersion). The pitting current, *i*_p_, shifted to higher values for the deformed areas after 3 h of immersion and continued to increase over time. Afterwards, an abrupt change in the current density was observed, which indicates the position of the pitting potential (*E*_pit_) [25,42,58]. The pitting potential for both undeformed samples has the same value of *E*_pit_ = −0.68 V/REF (Table 3). Slightly more positive pitting potentials were recorded for the deformed samples, with *E*_pit_ = −0.64 V/REF after 3 h of immersion. Increasing the immersion time to 6 h decreased the pitting potential of the deformed area to *E*_pit_ = −0.66 V/REF (after 6 h of immersion). The numerical values of pitting corrosion resistance (*R*_pit_) may be described by the difference between pitting potential and corrosion potential, *E*_pit_ and *E*_corr_ [59,60]

(2) $$R_{\mathrm{pit}}=\left|E_{\mathrm{corr}}-E_{\mathrm{pit}}\right|$$

Considering the data presented in Figure 6b, the smallest difference between *E*_corr_ and *E*_pit_ was calculated for the undeformed area of the sample for both immersion periods (*R*_pit_ = 0.16 after 3 h of immersion, and *R*_pit_ = 0.14 after 6 h of immersion), suggesting that the undeformed area is less-resistant to localized corrosion.

Regardless of immersion time, the deformed area of the alloy showed the greatest *R*_pit_ = 0.22V. The higher *R* and *i*_p_ values of the deformed areas were attributed to the weakened passivation properties [59,60].

The extrapolated from Tafel plot data show corrosion potentials of *E*_corr_ = −0.84 V/REF and *E*_corr_ = −0.82 V/REF for the undeformed areas immersed for 3 and 6 h, respectively. Corrosion potentials registered for the deformed areas had slightly lower values than the corrosion potentials of the undeformed areas, with values of *E*_corr_ = −0.88 V/REF and *E*_corr_ = −0.86 V/REF after 3 and 6 h of immersion, respectively (Table 3). To further view the overall corrosion behavior of the analyzed materials, LPR data were recorded after 3 and 6 h of immersion. The corrosion rates after 3 h of immersion were 1.8 mpy for the undeformed region, and almost twice as high (2.6 mpy) for the deformed region of the material (Table 3). After 6 h of immersion, the corrosion rate in the undeformed area increased slightly to 2.6 mpy, and the corrosion rate of the deformed area reached a significantly higher value of 9.5 mpy.

#### 4.2.2. SEM Observations after Immersion

**Figure 7:**
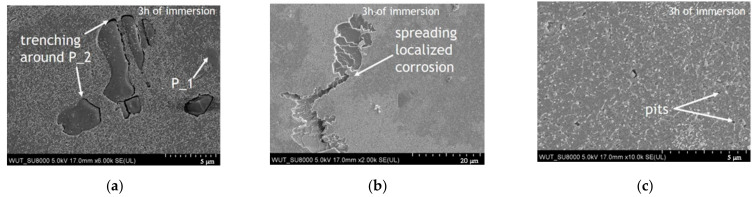

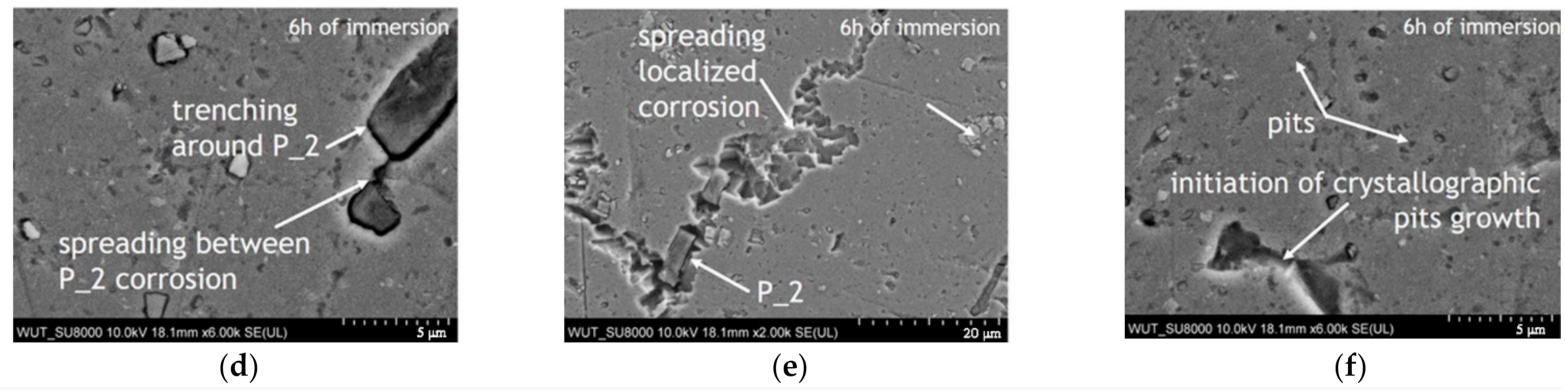
Post-corrosion observations of AA5754 in the undeformed area (A) after immersion under open-circuit conditions. Images taken after 3 h of immersion showing: (**a**), the propagation of crystallographically-grown pits, (**b**), trenching around Si-rich (P_2) particles, and (**c**) high-magnification image of tiny pits. Images taken after 6 h of immersion showing: (**d**), the propagation of crystallographically-grown pits, (**e**), trenching around Si-rich (P_2) particles, and (**f**) the formation of tiny pits.

**Figure 8:**
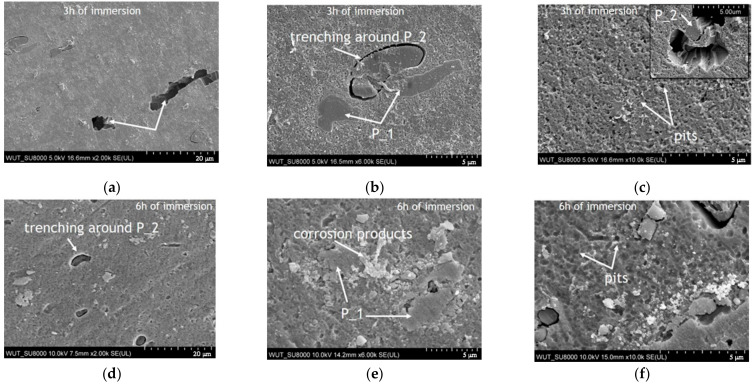
Post-corrosion observations of AA5754 in the deformed area (B) after immersion under open-circuit conditions. Images taken after 3 h of immersion showing: (**a**), the propagation of crystallographically-grown pits, (**b**), the trenching around Si-rich (P_2) particles, and (**c**), a high-magnification image of tiny pits. Images taken after 6 h of immersion showing: (**d**), trenching around Si-rich (P_2) particles, (**e**), corrosion products around Fe-rich (P_1) particles, and (**f**), the formation of tiny pits.

A comparison of the *E*_corr_ measurements under open-circuit conditions and surface analysis can be used to characterize the effect of immersion time on the microstructure-dependent corrosion processes. The corrosion morphologies of the undeformed and deformed areas after immersion in 0.5 M NaCl at pH 3 are presented in Figure 7 and Figure 8, respectively. The SEM images in Figure 7 and Figure 8 clearly show that the corrosion of AA5754 is a complex process that involves several microstructure-dependent corrosion mechanisms. At the beginning of the experiment, the main corrosion attack on both the undeformed and deformed samples occurred around the Si-rich intermetallic particles (marked as P_2 in Figure 7a and Figure 8b), while Fe-rich particles remained unreactive (marked as P_1 Figure 7a and Figure 8b). The local corrosion attack around the Si-rich constituents led to cavitation around the particles, as previously observed [61]. Moreover, SEM investigations clearly indicate that after 3 h of immersion, trenching was more intense around the particles on the deformed area (compare Figure 7a and Figure 8b). In both cases, trenching became more severe over time (Figure 7d and Figure 8d), and when two P_2 particles were relatively close to each other, trenching spread from one particle to the other, as shown in Figure 7d.

SEM observations confirmed that after immersion in 0.5 M NaCl (pH 3) trenching occurred around Si-rich particles over the entire analyzed sample surface. Besides intense local corrosion around Si-rich particles, after 3 h of immersion, highly-pronounced local pitting corrosion presented in the form of several superficial crystallographically-grown pits in the original and deformed areas of AA5754 (Figure 7b and Figure 8a) was formed. As the experiment duration increased, more crystallographically-grown pits were observed on the original material compared with the deformed area. Crystallographically-grown pits limited by perpendicular standing walls of the crystallographic lattice have been previously observed on Al and Al alloys, and the pit growth kinetics have been previously explained [62,63,64,65,66]. It was also demonstrated that in acidic chloride-containing solutions, the general mode of pit propagation is corrosion tunneling [62]. Considering the deformation effect on corrosion mechanisms on AA5754 after 3 h of immersion, minor differences were observed regarding the shape of crystallographically-grown pits (compare Figure 7a and Figure 8b), but not their amount. As the experiment duration increased, more crystallographically-grown pits were observed on the surface of the original material, and some underwent further lateral spreading, while on the deformed area after 6 h of immersion, only a few crystallographically-grown pits were observed, suggesting that no new pits were grown on the deformed area. The locally-formed crystallographic pits on the undeformed material spread laterally on the surface, whilst the pits on the deformed sample seemed to propagate into the depth of the material. Some pits were created around Si-rich particles (type P_2), as shown in Figure 7e and the inset in Figure 8c. In many areas of the observed samples, these kinds of pits were created after the trenches formed around P_2 particles.

Apart from the microgalvanic coupling between Si-rich particles and the matrix, or the large crystallographically-grown pits, very tiny metastable ellipsoidal pits formed on the surface of both areas (Figure 7c,f—undeformed areas after 3 and 6 h of immersion, respectively; Figure 8c,f—deformed areas after 3 and 6 h of immersion, respectively). These pits grew larger with the experiment duration (Figure 7f and Figure 8f); however, when comparing Figure 7c and Figure 8c, it is visible that more and deeper pits were created on the deformed area of the sample. Moreover, after 6 h of immersion, loosely-adhered corrosion products were formed on the deformed area of the sample, especially in the locations where two Fe-rich particles (P_1) were located relatively close to each other (Figure 8e).

## 5. Discussion

The results of this work show that the observed trenching around Si-rich coarse particles depends on their number and distribution, and the increased amount of these particles in the deformed area resulted in more localized corrosion initiation, which was one of the reasons for its lower corrosion resistance.

As demonstrated in this work, the corrosion that occurred around Si-rich particles in the deformed area of the material was more severe than the corrosion around the same type of particles in the undeformed area of AA5457. This phenomenon is related to the increased number of particles in the material after deformation and due to their mutual interaction with the matrix, particularly particle-dislocation interactions. In our opinion, the cumulative stress occurring during deformation was sufficient to cause particle fragmentation. The increased number of hard coarse particles provided more locations where trenching could occur, and the intensity of such trenching is related to particle-dislocation interactions. As is commonly known, the hard coarse particles are the places where deformation-induced dislocation movement is blocked, leading to the formation of a high concentration of dislocations around them [67].

The chemical composition of the coarse particles is also important, considering their corrosion behavior in chloride-containing solution. The Si-rich coarse intermetallics observed in AA5754 also contained Mg and Al. Previous reports have clearly indicated that the rapid dissolution of Mg from intermetallics occurs during exposure, which leads to the rapid dealloying of these particles [21,24,61,68,69]. The results of previous studies [68,69] explain the mechanism of Mg and Al dissolution from the coarse particles composed mainly of Mg and Si in Al-Mg-Si alloys. The results of this research show that at the beginning of immersion, the preferential and selective dissolution of Mg occurs, and thus, the Volta potential of the particle changes, which also changes the nature of Mg-containing particles from anodic to cathodic, forming a galvanic couple with the Al matrix. The results of this work show that the increased trenching around Si-rich particles in the deformed area was a result of the cracking of these particles induced by the cumulative stresses generated during deformation. During deformation, the coarse hard particles blocked the dislocation movement; therefore, a higher dislocation density was formed near such particles. As the locally-formed galvanic cells approach equilibrium, the cathodic particle forces the galvanic dissolution of the anodic matrix, leading to matrix corrosion. The increased dislocation density near the coarse particles promotes the corrosion reactions, leading to more intense trenching around the particles rich in Mg and Si. The reason for this is a higher dislocation density formed in the bent area of the sample, which promoted pit propagation in the depth due to their less-ordered structure [58,70]. This hypothesis is indisputably confirmed by the results presented in this work. It is also worth noting that Fe-rich particles remained unreactive throughout the entire experiment; however, their presence, especially when located relatively close to each other, may enhance corrosion product formation.

Another discrepancy that needs to be explained is that Aballe et al. [71] did not observe any relationship between the formation of crystallographic pitting and the existence of intermetallic particles, but Neetu et al. [42] claimed that pit initiation occurred at particles rich in Fe and Mn. We observed spontaneously-formed pits on the undeformed samples; however, in contrast to both works, our observations indicate that crystallographic pit growth is often initiated in the areas occluded by secondary particles enriched with Si. Moreover, this observation is supported by the fact that corrosion attack around Si-rich particles occurred before crystallographically-grown pits were formed. In a separate set of experiments, we observed trenching around Si-rich particles, but we did not observe any crystallographically-grown pits on the samples immersed for one hour in the same solution (data not published). Since pitting attack is considered to be an autocatalytic process in the active areas, it is reasonable to assume that the higher number of Si-rich coarse particles provided more sites were localized corrosion may be initiated. These places are prone to coalescence and form laterally-spreading local corrosion.

The second type of corrosion that occurred on the AA5754 alloy is microgalvanic corrosion between Si-rich nanoparticles and the alloy matrix. The observations showed that tiny pits were created around Si-rich nanoparticles, and the corrosion damage around them was more severe in the deformed area. Although the deformation-induced stress was not sufficient to change the size or distribution of the observed nanoparticles, our experiments clearly show that the nanoparticles in the deformed area with a high dislocation-stacking fault density around them promoted the formation of many deep tiny pits.

## 6. Conclusions

Based on the results of this work, the following conclusions can be drawn:

- The AA5754 alloy undergoes microstructure-dependent corrosion attack.
- The bending that was applied to obtain the desired shape of the design elements made from AA5754 affected the corrosion resistance of the alloy—the bent areas were more susceptible to corrosion than the original material.
- The lower corrosion resistance of the bent areas was related to the bending-induced microstructural changes, such as the increased density of Si-rich coarse particles and nanoparticles, the increased dislocation density around them, and their mutual interactions.

## Figures and Tables

**Table 1:** The chemical composition of 5754 alloy in mass% [48].

Si	Fe	Cu	Mn	Mg	Cr	Zn	Ti	Al
max	max	max	max		max	max	max	-
0.40	0.40	0.10	0.50	2.6–3.6	0.30	0.20	0.15	Bal.

**Table 2:** The mean surface area (*A*), standard deviation (*S*_D_), and number per 1 µm^2^ (*N*_A_) of coarse intermetallic particles P_1 and P_2.

AA5754	P_1			P_2		
Area	*A* (µm^2^)	*S*_D_	*N*_A_ (1 × µm^−2^)	*A* (µm^2^)	*S*_D_	*N*_A_ (1 × µm^−2^)
undeformed (A)	1.09	2.92	0.01	2.74	2.82	0.001
deformed (B)	0.59	1.68	0.02	2.33	3.89	0.01

**Table 3:** The corrosion potential (*E*_corr_), pitting potential (*E*_pit_), corrosion current density (*i*_corr_), and corrosion rate calculated after 3 and 6 h of immersion in naturally-aerated 0.5 M NaCl (pH 3) for the undeformed and deformed areas of AA5754.

Electrochemical Parameters	Sample Area and Immersion Time			
	Undeformed	Deformed	Undeformed	Deformed
	After 3 h of Immersion		After 6 h of Immersion	
*E*_corr_ (V/REF)	−0.84	−0.88	−0.82	−0.86
*E*_pit_ (V/REF)	−0.68	−0.64	−0.68	−0.66
*i*_corr_ (μA·cm^−2^)	18.6	34.1	31.6	32.7
Corrosion rate (mpy)	1.8	2.6	2.6	9.5

## Data Availability

Data available in a publicly accessible repository.
